# A comprehensive review on lncRNA LOXL1-AS1: molecular mechanistic pathways of lncRNA LOXL1-AS1 in tumorigenicity of cancer cells

**DOI:** 10.3389/fonc.2024.1384342

**Published:** 2024-07-29

**Authors:** Saghar Yousefnia

**Affiliations:** Department of Cell and Molecular Biology, Semnan University, Semnan, Iran

**Keywords:** long noncoding RNAs (lncRNAs), LOXL1-antisense RNA 1 (LOXL1-AS1), MicroRNA (miRNA), cancer, Tumorigenicity

## Abstract

Long non-coding RNAs (lncRNAs) are versatile RNAs that regulate various cellular processes, such as gene regulation, by acting as signals, decoys, guides, and scaffolds. A novel recognized lncRNA, LOXL1-antisense RNA 1 (LOXL1-AS1), is dysregulated in some diseases, including cancer, and acts as an oncogenic lncRNA in many types of cancer cells. Upregulation of LOXL1-AS1 has been involved in proliferation, migration, metastasis, and EMT, as well as inhibiting apoptosis in cancer cells. Most importantly, the malignant promoting activity of LOXL1-AS1 can be mostly mediated by sequestering specific miRNAs and inhibiting their binding to the 3´UTR of their target mRNAs, thereby indirectly regulating gene expression. Additionally, LOXL1-AS1 can decoy transcription factors and proteins and prevent their binding to their regulatory regions, inhibiting their mechanistic activity on the regulation of gene expression and signaling pathways. This review presents the mechanistic pathways of the oncogenic role of LOXL1-AS1 by modulating its target miRNAs and proteins in various cancer cells. Having information about the molecular mechanisms regulated by LOXL1-AS1 in cancer cells can open ways to find out particular prognostic biomarkers, as well as discover novel therapeutic approaches for different types of cancer.

## Introduction

Cancer is a challenging disease with increased rates of prevalence and mortality characterized by uncontrolled growth and loss of cell differentiation (1, 2). Cancer is recognized as the second leading cause of death all over the world (3). According to global cancer statistics for 2022, it is estimated that 9.7 million people died from cancer worldwide, and approximately 20 million new cases were diagnosed. Predictably, the number of cancer cases will reach 35 million by 2050 (4). It is characterized by high rates of proliferation, migration, invasion, metastasis, angiogenesis, and chemo/radiotherapy resistance, which are initiated and developed by genetic and epigenetic alterations. Numerous oncogenes and tumor suppressive genes regulate the malignant properties of cancer through various recognized molecular pathways (5). The most widespread types of cancer include breast, lung, colorectal, prostate, and skin cancer (6). Early cancer detection is critical for choosing the best treatment and optimizing therapeutic strategies (7). Advances in early detection, personalized medicine, and options for treatment have improved the prognosis of the disease. Knowledge about signaling pathways and genetic and epigenetic alterations associated with cancer can detect specific biomarkers suitable for targeted therapy (8). Recently, many studies have been conducted to develop new treatment approaches based on personalized medicine. Immunotherapy, phytochemicals, and other biomarker-specific targeted therapies are applied based on the specific molecular features in patients (8–10). Also, various nanostructure materials such as nanopolymers (polyethylenimine, polylactic-co-glycolic acid/PLGA, chitosans, collagen and gelatin), phytochemicals-based nanoparticles and inorganic materials (gold, diamond, silica, and ferric oxide) have been manipulated to design nanostructured carriers to deliver drugs into cancer cells and cancer stem cells more specifically and effectively (5, 11). However, cancer remains a significant global health challenge and a focus of ongoing research and medical advancements (7). There are many regulatory molecules, including long non-coding RNAs (LncRNAs), which are dysregulated in cancer and promote the malignant phenotypes of cancer (12).

LncRNAs are a group of untranslated RNA with a length size of more than 200 nucleotides (13). LncRNAs show complicated arrangements, allowing them to interact with DNA, RNA, and proteins. Despite not being involved in protein production, long non-coding RNAs (lncRNAs) exhibit acute roles in various cellular processes, including gene regulation at both the transcriptional and post-transcriptional levels, regulating protein activity, controlling protein localization, facilitating genomic imprinting, modifying chromatin, and influencing mRNA stability (13–15). They can act as signals, decoys, guides, and scaffolds (16, 17), thus affecting a variety of biological processes, such as cell proliferation, differentiation, and apoptosis (13, 14, 18). Most importantly, lncRNAs are referred to as competing endogenous RNAs (ceRNAs) by sequestering miRNAs. LncRNAs can sponge specific miRNAs and prevent them from binding to 3´UTR of their target mRNAs, thereby indirectly modulating gene expression (19). Dysregulation of lncRNAs has been associated with various diseases, including neurodegenerative disorders (16, 20), cardiovascular disease (21) and cancer (22).

Recently, RNA sequencing and genetic analysis have identified a novel lncRNA, lncRNA LOXL1-antisense RNA 1 (LOXL1-AS1) encoded on the opposite strand of LOXL1 (23). The expression of LOXL1-AS1 is altered in cellular stress responses, oxidative stress and cyclic mechanical stress (23). LOXL1-AS1 is also dysregulated in certain diseases such as atherosclerosis (24, 25), osteoarthritis (26), periodontitis (27) and postmenopausal osteoporosis (28). Furthermore, overexpression of LOXL1-AS1 has been confirmed in a range of cancers, which predicts a poorer prognosis, increased risk of cancer recurrence, and higher likelihood of metastasis. LOXL1-AS1 has also been proposed as a potential prognostic biomarker in patients with cancer (29). LOXL1-AS1, as an oncogene lncRNA, plays crucial roles in tumorigenesis and development of various types of cancer, including ovarian cancer (30), gastrointestinal cancer (31, 32), lung cancer (33), hepatocellular carcinoma (34), breast cancer (35), prostate cancer (36) and others. Mechanistically, this lncRNA has been involved in cell proliferation, apoptosis, migration, metastasis and epithelial-mesenchymal transition (EMT) of these types of cancer cells through its target miRNAs and indirectly regulates the expression of specific target genes of miRNAs.

LOXL1-AS1 has been found to function as a ceRNA or a sponge for miRNAs (24, 31, 37). This means that LOXL1-AS1 has binding sites for specific miRNAs, enabling it to effectively sequester and prevent them from binding to their target mRNAs (38). In other words, LOXL1-AS1 and mRNA share the same miRNA binding sites, which are recognized as miRNA response elements (MREs), making them competitive to bind miRNAs (24, 39). By acting as a sponge for miRNAs, LOXL1-AS1 can influence gene expression by regulating the availability of miRNAs to control the expression of other genes. This regulatory function can have implications for various cellular processes, including development, disease progression, and cellular homeostasis (38, 40).

This review aims to elucidate the mechanistic pathways underlying the oncogenic role of LOXL1-AS1 by regulating its target microRNAs and proteins in various types of cancer cells. Understanding the molecular mechanisms governed by lncRNA LOXL1-AS1 in cancer cells may provide opportunities for identifying specific prognostic biomarkers and developing novel therapeutic strategies for diverse types of cancer.

## LOXL1-AS1 in various cancers

### Ovarian, cervical and endometrial cancer

**Figure 1:**
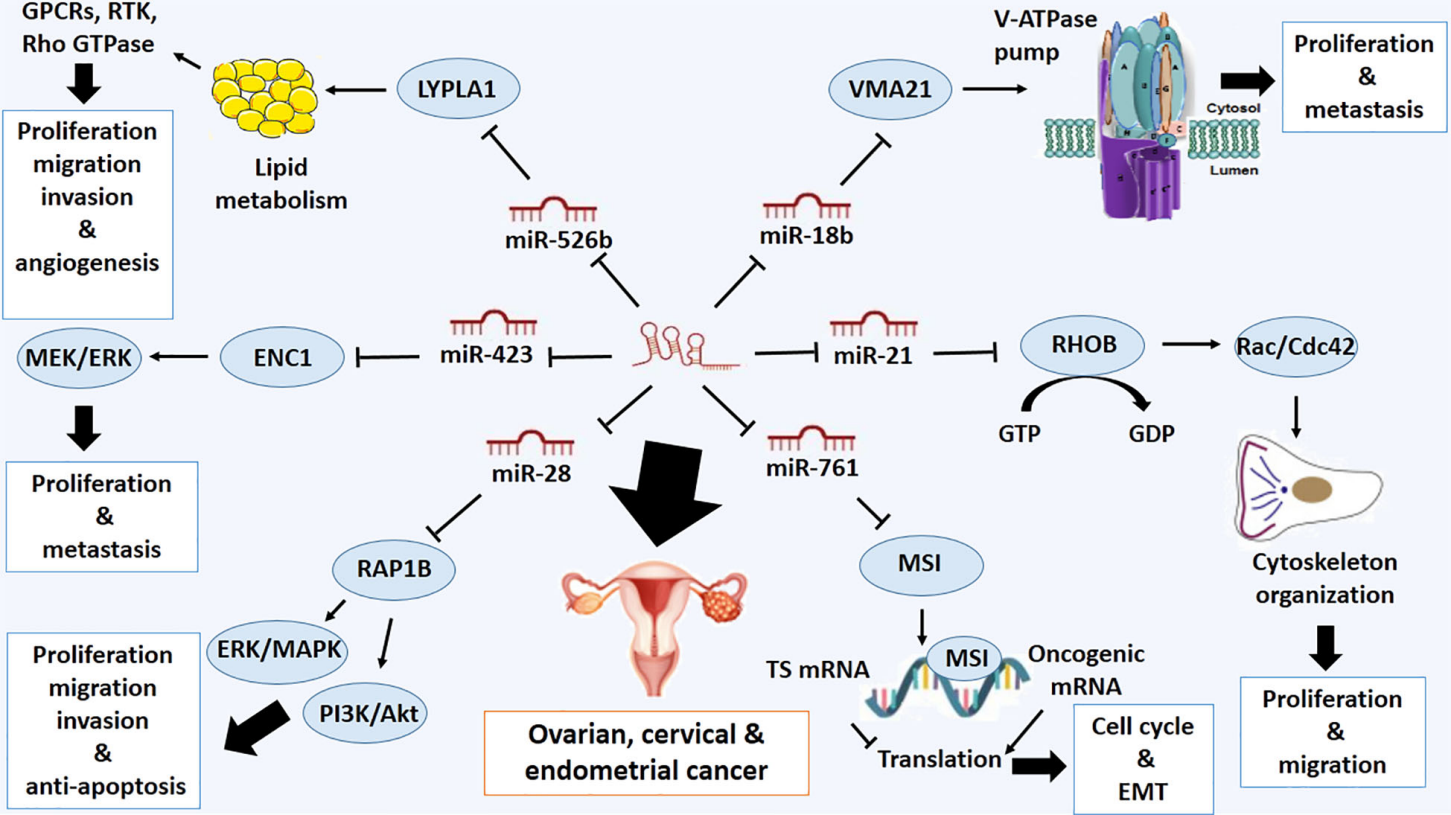
Molecular mechanistic activity of LncRNA LOXL1-AS1 in ovarian, cervical and endometrial cancer.

The gynecologic cancers, ovarian, cervical and endometrial cancer are three types of cancer that affect the adjacent organs of the female reproductive system. Endometrial cancer has the highest relative survival rate (RSR), while ovarian cancer has the lowest (41). Serum LOXL1-AS1 has been introduced as a diagnostic and prognostic marker to predict ovarian cancer patients with high sensitivity (65.3%) and specificity (68.2%) (42). Also, LOXL1-AS1 overexpression is associated with advanced stages of tumor and metastasis with poor clinical outcome (42). LOXL1-AS1 can regulate malignant phenotypes of ovarian cancer and decrease the apoptotic rate of ovarian cancer cells by targeting several miRNAs (30, 43). miR-761 can be considered a direct target of LOXL1-AS1 and miR-761 inhibitor can reduce the oncogenic role of LOXL1-AS1 in ovarian cancer cells (30). It has been previously verified that Musashi1 (MSI1) is a target of miR-761 in ovarian cancer. MSI1 is an RNA-binding protein that plays crucial roles in various biological processes, including nervous system development, stem cell fate determination, and tumorigenesis, due to its key regulatory function in translation (44).. Additionally, it plays a crucial role in cancer development and progression by regulating the expression of key oncogenes and tumor suppressor genes. MSI1 has been shown to promote cancer cell proliferation, invasion, and metastasis through its ability to stabilize mRNA transcripts that encode factors involved in cell cycle and EMT (45). It also binds to the cell cycle checkpoint and apoptosis regulators, such as p21, p27, and p53, and inhibits their translation (45) (**Figure 1**).

Also, LOXL1-AS1 can bind to miR-18b-5p and miR-18b-5p targets Vacuolar ATPase Assembly Factor (*VMA21*) in ovarian cancer. Silencing LOXL1-AS1 and upregulating miR-18b-5p can inhibit the proliferation and metastasis of ovarian cancer cells by targeting *VMA21* (43). The VMA21 protein plays a crucial role in forming V-ATPase complexes within cells. V-ATPases act as proton pumps responsible for modulating the acidity levels within intracellular compartments, including lysosomes and the external environment. The proper functioning of these complexes is indispensable for various cellular processes, including protein degradation, membrane transport, and signal transduction pathways. Furthermore, research has demonstrated that aberrant expression of V-ATPases and their assembly factors contribute to elevated tumor acidity, a phenomenon closely tied to cancer progression and treatment resistance. Notably, VMA21’s involvement in altering pH dynamics facilitates the survival and proliferation of cancerous cells within the acidic tumor microenvironment (46) (**Figure 1**).

Also, it has been reported that there is a positive correlation between the expressions of LOXL1-AS1 and RHOB. LOXL1-AS1 overexpression results in upregulation of RHOB (47). RHOB is a member of the Rho family of small GTPases, which plays a role in regulating the organization of the actin cytoskeleton, cell adhesion, migration, and proliferation of cancer cells by activating Rac/Cdc42 (48). It has been shown that RHOB is a direct target of miR-21, which is downregulated in cervical squamous cell carcinoma (CSCC). Predicting that LOXL1-AS1 can interact with miR-21. However, there is no significant evidence on the reciprocal effect of LOXL1-AS1 expression on miR-21 expression (47) (**Figure 1**).

One of the other miRNA targets of LOXL1-AS1 is miR-526b-5p, which is down-regulated in cervical cancer cells. LOXL1-AS1 depletion suppresses proliferation, migration, invasion, and angiogenesis of this type of cancer cells through downregulating Lysophospholipase 1 (LYPLA1), a direct target of miR-526b-5p (49). LYPLA1 is crucial in lipid metabolism and signal transduction in living organisms. This enzyme is responsible for the hydrolysis of lysophospholipids, which are involved in various signaling pathways as second messengers and regulate cell proliferation, migration, and survival (50). By cleaving lysophospholipids into smaller components, lysophospholipase can impact the overall balance of these signaling molecules and influence cellular responses, lipid digestion, cell membrane remodeling and lipid-mediated signaling pathways (49, 50). Lysophospholipids exert their effects by binding to specific G-protein coupled receptors and receptor tyrosine kinases on the cell surface, which leads to the activation of downstream signaling cascades (51). One key pathway mediated by Lysophospholipids is the Rho-GTPase pathway, which regulates actin cytoskeleton dynamics and cell motility. Additionally, Lysophospholipids can activate various intracellular signaling molecules, such as protein kinases and transcription factors, to modulate gene expression and promote cell growth (51) (**Figure 1**). The other target of LOXL1-AS1 is miR-423–5p in cervical cancer. miR-423–5p, the downstream of LOXL1-AS1, directly targets ectodermal-neural cortex 1 (*ENC1*). Importantly, LOXL1-AS1 increases *ENC1* expression through sequestering miR-423–5p in cervical cancer. Besides, miR-423–5p acts as a tumor-inhibitor while ENC1 works as a tumor-facilitator in cervical cancer, promoting proliferation and metastasis of this type of cancer cells through activation of the mitogen-activated protein kinase/extracellular signal-regulated kinase (MEK/ERK) and MAPK pathway (52). ENC1 activates the MAPK signaling pathway, which involves various cellular processes, including proliferation, differentiation, and survival. All of these processes are dysregulated in cancer cells. By activating this signaling cascade, ENC1 promotes tumor growth and invasion by facilitating cell proliferation and inhibiting apoptosis (53) (**Figure 1**).

LOXL1-AS1 is also upregulated in endometrial cancer (EC) cells, and its knockdown decreases cell proliferation, migration, and invasion of EC, while promoting apoptosis. The oncogenic roles of LOXL1-AS1 are mediated by the upregulation of Ras-related protein 1B (RAP1B), which is one of the direct targets of microRNA-28–5p (54). RAP1B is a small GTPase protein that is involved in various cellular processes, including cell growth, cell division, cell adhesion, cell movement, and intracellular protein trafficking (55).. RAP1B promotes the activation of pathways such as phosphatidylinositol 3-kinase/Akt and extracellular signal-regulated kinase/mitogen-activated protein kinase (ERK/MAPK), leading to enhanced cell proliferation and survival (56). Since RAP1B can be downregulated by microRNA-28–5p, it can be recognized as a potential tumor-suppressive microRNA and direct target of LOXL1-AS1 (54) (**Figure 1**).

Therefore, it may propose new considerations about the molecular mechanism of LOXL1-AS1 in gynecologic cancers to develop novel therapeutic approaches for this type of cancer.

### Gastrointestinal cancer

**Figure 2:**
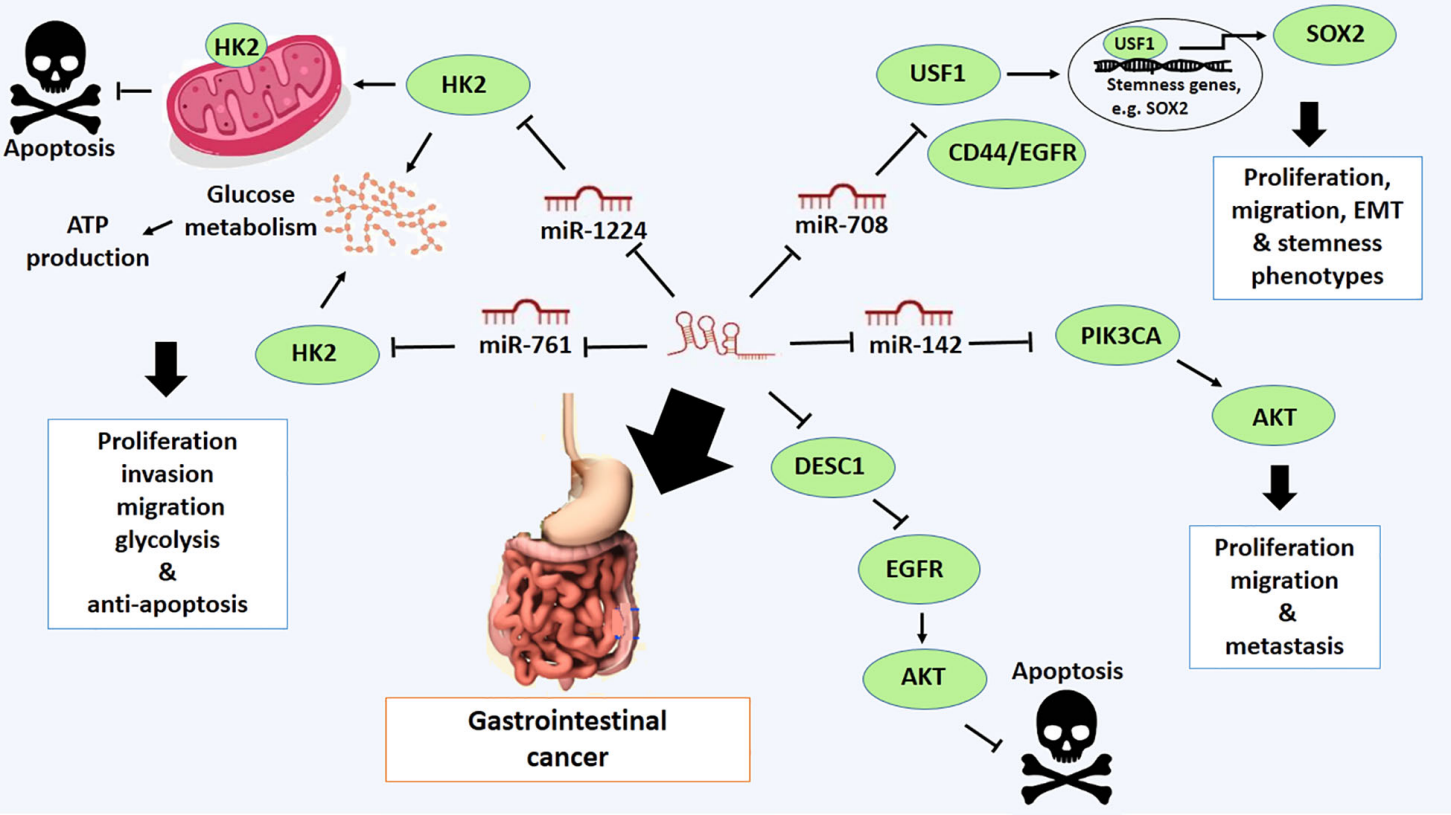
Molecular mechanistic activity of LncRNA LOXL1-AS1 in gastrointestinal cancer.

Gastrointestinal cancer is a group of cancers that involve the digestive system. These are gastric, colorectal and esophagus. It has the highest rate of incidence and mortality in developing and developed countries (57). Mainly, LOXL1-AS1 is highly expressed in gastric cancer, leading to proliferation, migration, EMT and stemness phenotypes of gastric cancer. It positively regulates upstream transcription factor 1 (USF1), a critical factor in promoting the expression of stemness genes, including SOX2. Furthermore, USF1 is negatively regulated by miR-708–5p, suggesting LOXL1-AS1 implicates the oncogenic activity by targeting miR-708–5p in gastric cancer (31). In addition, overexpression of LOXL1-AS1 is associated with short general survival time and malignant phenotypes of this type of cancer. LOXL1-AS1 is also linked with downregulation of miR-142–5p and upregulation of PIK3CA, suggesting a LOXL1-AS1/miR-142–5p/PIK3CA axis in the progression and development of gastric cancer (58) (**Figure 2**).

The oncogenic role of LOXL1-AS1 has also been confirmed in colorectal cancer. The high expression level of LOXL1-AS1 is correlated with the development of colorectal cancer through promoting proliferation, migration and invasion (32). The mechanistic activity of LOXL1-AS1 is mediated by a reduction in the expression of miR-708–5p and an enhancement in the CD44/EGFR expression. LOXL1-AS1 implicates targeting miR-708–5p and thereby promotes the malignant phenotypes of colorectal through activating CD44-EGFR signal pathway (32). CD44 is well-known as a hyaluronic acid receptor highly expressed on the cell surface of malignant cancer cells. It can be involved in cell-cell interaction, cell adhesion, and migration. It can also bind to other ligands, including osteopontin, collagens and matrix metalloproteases (MMPs). CD44, along with epithelial growth factor receptor (EGFR), may regulate metastasis and cell signaling pathways, including the Ras-MAPK and PI3K/Akt pathways, which are implicated in the regulation of malignant phenotypes of cancer cells, characterized by cellular processes such as cell adhesion, migration, invasion, and epithelial-to-mesenchymal transition (EMT) (59) (**Figure 2**).

The other targets of LOXL1-AS1 are miR-1224–5p and miR-761, targeting HK2 in colorectal cancer (60). LOXL1-AS1 downregulation suppresses the expression of HK2 and inhibits cell proliferation, invasion, migration, and glycolysis, while promoting apoptosis. However, these effects are inverted by suppression of miR-1224–5p and miR-761 (60). The HK2 protein, also known as hexokinase 2, is an enzyme that plays a critical role in the first step of glucose metabolism. It catalyzes the conversion of glucose to glucose-6-phosphate, which is a primary step in glycolysis and glycogen synthesis (61). HK2 is particularly important in cancer cells, where it is often overexpressed and contributes to the characteristic increased glucose metabolism of many cancer types. This upregulation allows cancer cells to maintain high levels of ATP production even under low oxygen conditions through aerobic glycolysis, also known as the Warburg effect (61). This overexpression of HK2 is thought to provide a growth advantage to cancer cells by allowing them to more efficiently utilize glucose for energy and mass production (61). Additionally, HK2 protects cancer cells from apoptosis by docking to mitochondria and inhibits cell death by regulating the mitochondria-mediated intrinsic pathway (61). Therefore, LOXL1-AS1 has been contributed to colorectal development via regulating LOXL1-AS1/miR-1224–5p/miR-761/HK2 axis (60) (**Figure 2**).

The high expression of LOXL1-AS1 has also been verified in esophageal squamous cell carcinoma (ESCC). Overexpression of LOXL1-AS1 promotes cell proliferation, migration, and invasion, while also inhibiting apoptosis (62). The oncogenic activity of LOXL1-AS1 is mediated by its primary downstream target, the DESC1 protein, which is differentially expressed and downregulated in esophageal squamous cell carcinoma (ESCC) (62). DESC1 is a Type II transmembrane serine protease recognized as a novel tumor suppressor protein. It induces apoptosis in response to apoptotic stimuli by modulating the EGFR/AKT signaling pathway. Mechanistically, DESC1 cleaves EGFR through proteolytic activity and inhibits AKT1 activation, thereby sensitizing cells to apoptosis. Consequently, downregulation of DESC1 can be attributed to the malignant characteristics of cancer cells (63) (**Figure 2**).

Furthermore, taken together, LOXL1-AS1 emerges as a promising target for recommending novel advanced therapeutic strategies for both diagnosis and treatment of gastrointestinal cancer.

### Lung cancer and laryngeal carcinoma

**Figure 3:**
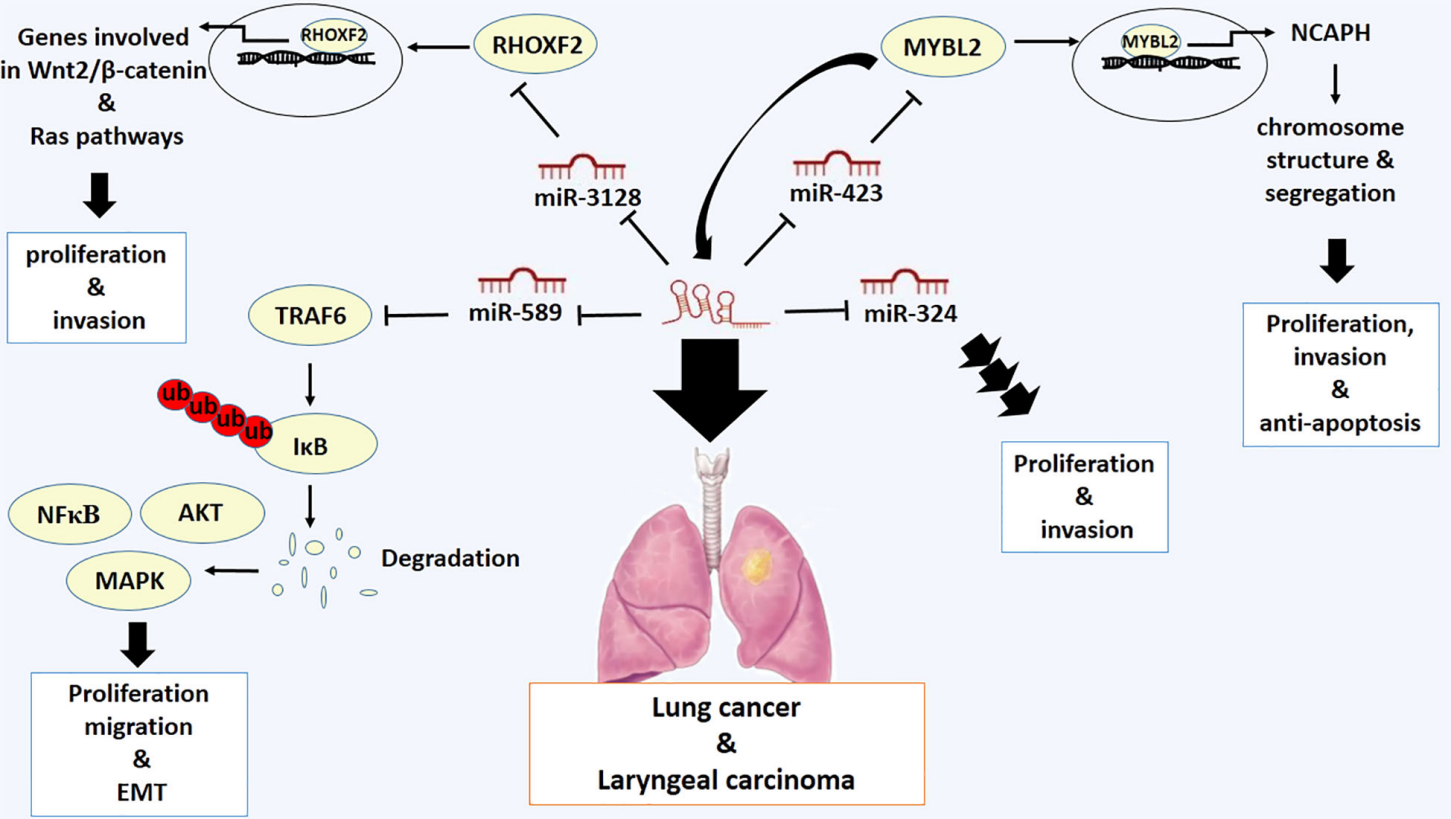
Molecular mechanistic activity of LncRNA LOXL1-AS1 in lung cancer and laryngeal carcinoma.

Lung cancer is the second most prevalent type of cancer, with 2 million new cases and 1.8 million deaths (64). The evidence demonstrates that LOXL1-AS1 is highly expressed in non-small-cell lung cancer (NSCL) cell lines and tissues. Overexpression of LOXL1-AS1 promotes cell proliferation by inducing Ki-67 and Cyclin D1 expression, drives invasion by inducing N-cadherin and Vimentin expression, and suppresses E-cadherin expression in NSCL (33). Low expression of miR-324–3p in these cancer cells has indicated that the oncogenic activity of LOXL1-AS1 may be mediated by miR-324–3p in this type of cancer cells. miR-324–3p restoration decreases the proliferative and oncogenic function of LoxL1-AS1, proposing that LOXL1-AS1 increases proliferation and invasion of NSCL cells through targeting miR-324–3p (33). High expression of LOXL1-AS1 is also linked with developed stages and metastasis of NSCL cells. LOXL1-AS1 and Rhox homeobox family member 2 (*RHOXF2*) are highly expressed and miR-3128 is expressed at low levels in NSCL cells. LOXL1-AS1 acts as a sponge that targets miR-3128 to promote *RHOXF2* expression, thereby promoting metastasis of this type of cancer cells (65). The RHOXF2 protein is a member of the RHOX family of homeobox genes, which encode transcription factors involved in regulating the expression of genes related to the development of cancer (66). Mechanistically, RHOXF2 promotes cancer cell proliferation and invasion by regulating key signaling pathways, including the Wnt2/β-catenin pathway, which is involved in tumor progression (67). Additionally, RHOXF2 has been found to interact with other oncogenes and tumor suppressor genes to drive malignant transformation (66). It is supposed that the expression of genes repressed or increased by RHOXF2 may be involved in the Ras pathway (68) (**Figure 3**).

In addition, the proliferative and anti-apoptotic activities of LOXL1-AS1 in lung cancer are mediated by the upregulation of MYBL2, a Myb-related protein, which occurs as a result of sponging miR-423–5p (38). The MYBL2 protein, also known as B-Myb, is a transcription factor involved in regulating the cell cycle. It is also involved in the control of cell proliferation, differentiation and apoptosis. The MYBL2 protein is involved in several cellular processes, including DNA replication and repair, and it is primarily active during the G2 and M phases of the cell cycle (69). It has been reported that Non-SMC Condensin I Complex Subunit H (NCAPH) is a responsive downstream target gene of MYBL2 protein (70). NCAPH) plays a crucial role in cancer development and progression. As part of the condensin complex, NCAPH is involved in regulating chromosome structure and segregation during cell division. Therefore, overexpression of NCAPH has been linked to increased tumor growth, invasion, and metastasis, while its downregulation can impair cell proliferation and induce apoptosis in cancer cells (71). More importantly, it has been discovered that MYBL2 protein interacts with LOXL1-AS1 promoter and promotes the LOXL1-AS1 expression, demonstrating a positive feedback loop of LOXL1-AS1/miR-423–5p/MYBL2 in lung adenocarcinoma (38) (**Figure 3**).

Laryngeal carcinoma is also the second type of cancer after lung cancer that involves the upper aerodigestive tract (72). The oncogenic role of LOXL1-AS1 has also been verified in laryngeal carcinoma. LOXL1-AS1 has been implicated in cell proliferation, migration, and EMT of this type of cancer. LOXL1-AS1 stimulates the expression of tumor necrosis factor receptor-associated factor 6 (*TRAF6*) as a sponge targeting miR-589–5p. Additionally, it has been shown that knockdown of miR-589–5p drives the development of tumors by promoting the expression of TRAF6 (73). TRAF6 is an unconventional E3 ubiquitin ligase and a key mediator of ubiquitin-dependent NF-κB, MAPK, and AKT activation, which are essential pathways involved in regulating cell survival, proliferation, and inflammation (74). Therefore, it has been validated that LOXL1-AS1 promotes the malignancy in laryngeal carcinoma by modulating the miR-589–5p/TRAF6 pathway (73) (**Figure 3**).

In summary, understanding the molecular mechanisms of LOXL1-AS1 activity can provide novel, applicable tools for detecting and combating lung and laryngeal carcinomas.

### Hepatocellular carcinoma and cholangiocarcinoma

**Figure 4:**
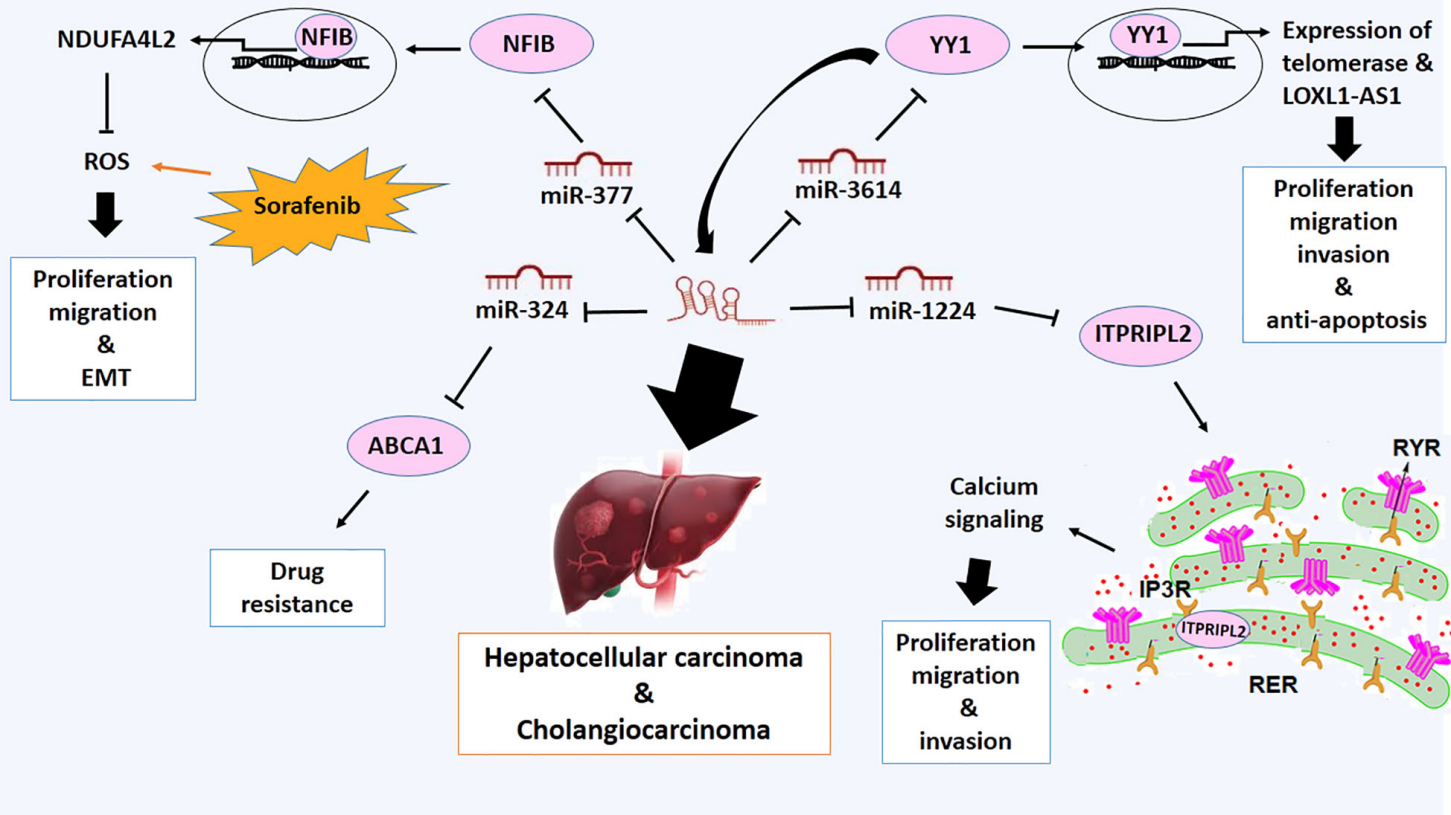
Molecular mechanistic activity of LncRNA LOXL1-AS1 in hepatocellular and cholangiocarcinoma.

Hepatocellular carcinoma (HCC) or liver cancer commonly arises in cases with chronic liver diseases and cirrhosis as a result of hepatitis B or C infection (75). LOXL1-AS1 is overexpressed in hepatocellular carcinoma and stimulates the proliferation, migration, and metastasis of these types of cancer cells (76, 77). Functionally, the metastatic activity of LOXL1-AS1 can be attributed to the increased expression of matrix metalloproteinase (MMP)-2 and MMP-9 proteins (76). It has also been verified that silencing LOXL1-AS1 induces G0/G1 phase cell cycle arrest, which is mediated by a reduction in the expression of Cdc2, Cdc25A, and cyclin B1 proteins. Consequently, overexpression of LOXL1-AS1 can promote the proliferation, migration, and invasion of HCC cells (76). Furthermore, LOXL1-AS1 acts as a ceRNA to elevate inositol 1, 4, 5-trisphosphate receptor-interacting protein-like 2 (ITPRIPL2) level through targeting miR-1224–5p and exhibit the malignant phenotypes of HCC via activating AKT pathway, thereby playing an oncogenic role in HCC (34). ITPRIPL2 is a protein involved in calcium signaling, regulating intracellular calcium levels. Calcium signaling is crucial for regulating various cellular processes such as proliferation, migration, and invasion in cancer cells. However, its specific role in cancer may not be well-established or widely documented (78). It has been reported that ITPRIPL2 modulates calcium release from intracellular stores by interacting with inositol 1,4,5-trisphosphate receptors (IP3Rs), leading to dysregulated calcium levels that promote tumor growth and metastasis (79) (**Figure 4**).

One of the other miRNA targets of LOXL1-AS1 is miR-3614–5p, which is down-regulated in HCC. Downregulation of miR-3614–5p due to upregulation of LOXL1-AS1 promotes proliferation, migration, and invasion and inhibits apoptosis (80). Furthermore, Yin Yang 1 (YY1) is recognized as a direct target of miR-3614–5p, which is upregulated in HCC. YY1 depletion can suppress malignant phenotypes of HCC. There is a positive feedback loop between YY1 and LOXL1-AS1 expressions (80) (**Figure 4**). YY1 acts as a transcription factor to activate the expression of LOXL1-AS1 and plays a role in regulating the proliferation, apoptosis, and differentiation of hepatocellular carcinoma (HCC) cells (81). Additionally, YY1 has been associated with regulating telomerase, a crucial enzyme essential for maintaining telomere length and ensuring replicative immortality in cancer cells (81).

Moreover, it has been verified that LOXL1-AS1 sponges miR-377–3p, the other direct target of LoxL1-AS1, and miR-377–3p acts as an upstream direct regulator of nuclear factor I B (*NFIB*) gene in liver cancer (82). *NFIB* gene encodes a transcription factor implicated in regulating genes related to cell proliferation, differentiation and malignant phenotypes of hepatocellular carcinoma (83). NFIB can bind with the promoter of a complex I inhibitor NDUFA4L2 and promote its expression (84). Upregulation of NDUFA4L2, as a redox modulator, inhibits reactive oxygen species accumulation induced by drugs like sorafenib. NFIB may be able to protect liver cancer cells from oxidative stress and promote their survival in the presence of chemotherapy (84). Therefore, It is proposed that LoxL1-AS1/miR-377–3p/NFIB axis promotes proliferation, migration and EMT of liver cancer (82) (**Figure 4**).

Cholangiocarcinoma (CCA) is the other type of hepatic malignancy. It is a type of cancer that forms in the bile ducts. It is a relatively rare and aggressive form of cancer; however, the occurrence and mortality rates of CCA are growing worldwide (85). Upregulation of LOXL1-AS1 has also been observed in cholangiocarcinoma (CCA) and has been associated with lymph node invasion, advanced disease stages, increased cell proliferation, enhanced cell migration, and attenuation of apoptosis (37). Mechanistically, LOXL1-AS1 interacts with miR-324–3p and abolishes the tumor suppressor function of miR-324–3p. On the other hand, miR-324–3p can target ATP-binding cassette transporter A1 (ABCA1), which is implicated in the efflux of drugs from cancer cells, making them resistant to chemotherapy (37). Reduction in the expression of LOXL1-AS1 suppresses the expression of ABCA1 and suppresses malignant features and drug resistance of CCA (37) (**Figure 4**).

Taken together, it suggests a promising prognostic and diagnostic biomarker to identify as well as providing a novel therapeutic approach for liver cancer and cholangiocarcinoma.

### Breast cancer

**Figure 5:**
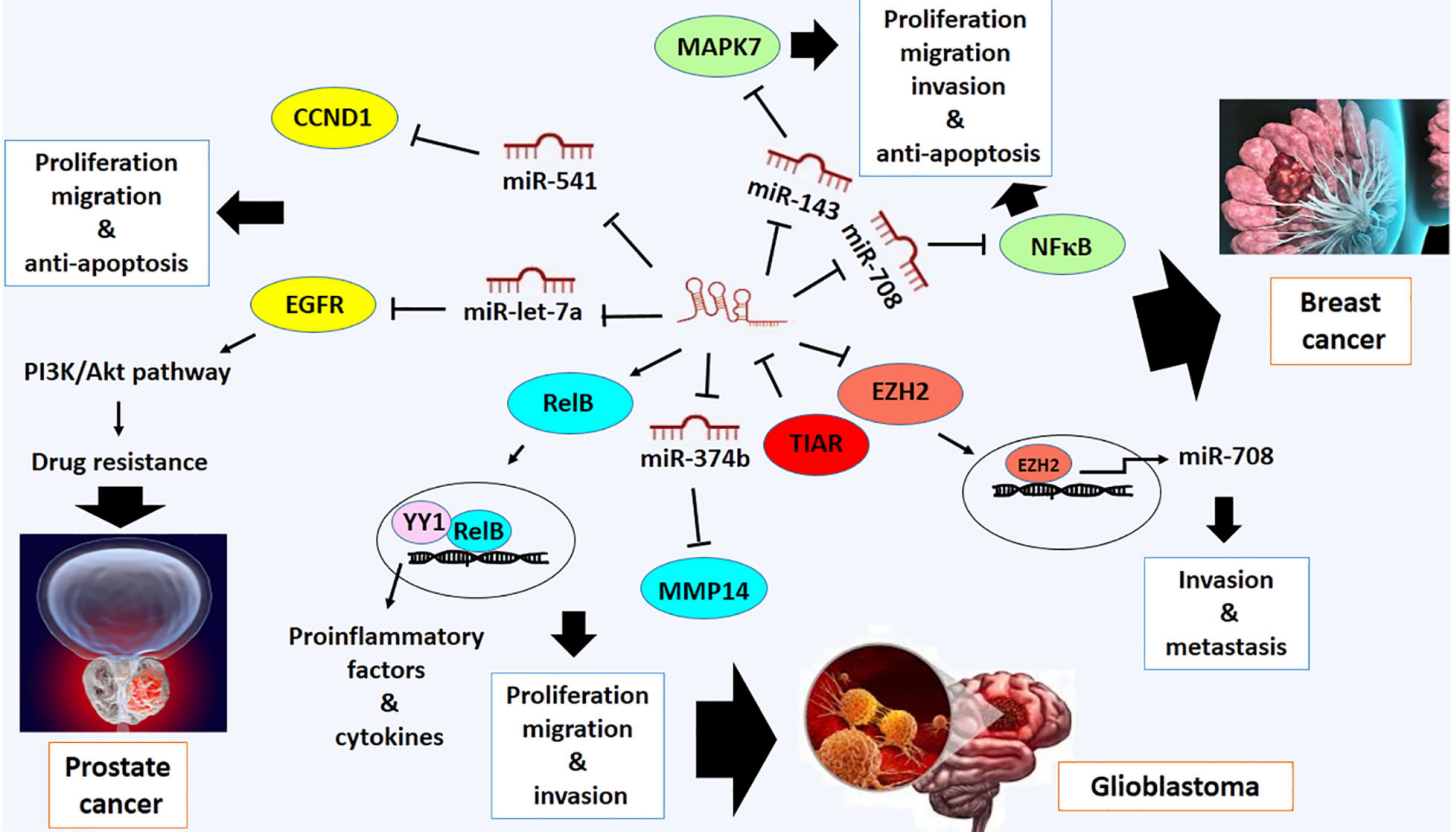
Molecular mechanistic activity of LncRNA LOXL1-AS1 in breast cancer, prostate cancer and glioblastoma.

Breast cancer is one of the most prevalent types of cancer worldwide, with a high mortality rate among women. It accounts for 25% of all cancers diagnosed in females and 15% of all cancer-related deaths (86). Deregulation of LOXL1-AS1 has also been observed in breast cancer. LOXL1-AS1 promotes cell proliferation, migration, and invasion while also inhibiting apoptosis in breast cancer cells (35). The malignant-promoting activity of LOXL1-AS1 may be mediated by the downregulation of miR-143–3p. It has been exhibited that LOXL1-AS1 directly targets miR-143–3p in this type of cancer cells (35). It has been previously shown that miR-143–3p plays a tumor-suppressive role in breast cancer by targeting MAPK7, a member of the MAP kinase family, which promotes signaling pathways involved in cell proliferation and anti-apoptosis in breast cancer (87) (**Figure 5**). Furthermore, overexpression of LOXL1-AS1 is associated with the stage of tumor and metastasis of this type of cancer (88).

One of the other direct targets of LOXL1-AS1 is miR-708–5p, which is downregulated in breast cancer (88). Mechanistically, miR-708–5p inhibits NF-κB activity, which is implicated in invasion and metastasis of breast cancer (88). NF-κB is a pro-inflammatory transcription factor that expresses IL-6 and cytokines involved in regulating proliferation, invasion, metastasis, and anti-apoptosis in breast cancer (89) (**Figure 5**). Additionally, LOXL1-AS1 interacts with the Enhancer of Zeste Homolog 2 (EZH2) transcription factor to suppress the transcription of miR-708–5p, which is regulated by the EZH2 protein (88). Therefore, the invasive and metastatic roles of LOXL1-AS1 may be mediated by blocking the expression of miR-708–5p and targeting it (88) (**Figure 5**). It provides a novel therapeutic strategy against breast cancer and improves understanding of the molecular mechanism of breast cancer development.

### Prostate cancer

Prostate cancer is the most common type of cancer among men worldwide. It ranks fifth among cancers involving men, and its incidence rate is increasing in both developing and developed countries (90). It has been reported that LOXL1-AS1 is also overexpressed in prostate cancer. LOXL1-AS1 has been involved in cell cycle progression and proliferation of this type of cancer (36). The molecular function of LOXL1-AS1 is mediated by the upregulation of cyclin D1 (CCND1), a cell cycle-promoting actor, which results from targeting miR-541–3p (36). miR-541–3p is a tumor suppressor miRNA recognized as a negative regulator of *CCND1* through binding to 3´UTR of *CCND1* and is downregulated in prostate cancer (36) (**Figure 5**).

Recently, research has shown that long non-coding RNA (lncRNA) LOXL1-AS1 has also been implicated in doxorubicin-resistant activity of prostate cancer. Drug resistant activity of prostate cancer may be modulated by upregulation of LOXL1-AS1 and EGFR and downregulation of miR-let-7a-5p, predicting miR-let-7a-5p and EGFR are negatively regulated by LOXL1-AS1 and miR-let-7a-5p, respectively (91). It is supposed that drug resistance activity of prostate cancer is due to overactivity of the PI3K/Akt pathway mediated by overexpressed EGFR (92). In addition, the upregulation of LOXL1-AS1 and EGFR has been implicated in promoting cell proliferation, enhancing cell migration, and inhibiting apoptosis in this type of cancer cells. It may provide a novel potential strategy of treatment for patients with drug-resistant prostate cancer (91) (**Figure 5**).

### Glioblastoma

Glioblastoma is a common type of brain tumor with a highly aggressive phenotype brain tumor. It estimates to affect less than 2% of all primary tumors; however, it is responsible for approximately 7% of deaths caused by cancer (93). Unfortunately, despite extensive research on these tumors, the survival rate for patients with brain tumors is meager, reflecting the lack of new treatment options for patients (93). It has also been reported that LOXL1-AS1 modulates tumor progression in glioblastoma (94). The experimental evidence has shown that increased expression of LOXL1-AS1 is linked to malignant biological processes, including the development of a mesenchymal phenotype in glioblastoma, by regulating the NF-κB signaling pathway (94). Down-regulating LOXL1-AS1 leads to inhibition of the NF-kB pathway by decreasing RelB expression with an unknown mechanism (94). RelB protein is a subunit of the NF-κB family and plays a key role in the regulation of NF-κB family members, which are transcription factors (95). It promotes the expression of pro-inflammatory factors and cytokines in glioblastoma. Mechanistically, the interaction between RelB and transcription Factor YY1 initiates specific gene expression programs in glioblastoma cells (96) (**Figure 5**).

Additionally, LOXL1-AS1 is a sponger of miR-374b-5p. Downregulation of miR-374b-5p due to LOXL1-AS1 overexpression promotes proliferation, migration, invasion and vasculogenic mimicry (VM) in glioma (97). The tumor suppressor role of miR-374b-5p can be mediated by targeting MMP14 (97) (**Figure 5**). Also, it has been confirmed that the expression of LOXL1-AS1 may be modulated by TIA-1-related protein (TIAR) (97). The TIAR protein is an RNA-binding protein that contributes to the regulation of gene expression at the post-transcriptional level. In cancer, TIAR is introduced as a regulator of various processes, including apoptosis, cell cycle, and response to cellular stress (97). TIAR downregulates the expression of LoxL1-AS1 by destabilizing LOXL1-AS1, suggesting TIAR with LOXL1-AS1 regulates VM in glioma through the TIAR/LOXL1-AS1/miR-374b-5p/MMP14 axis (97) (**Figure 5**). Therefore, these data may introduce potential targets for diagnosing and treating glioma.

### Other types of cancer

**Figure 6:**
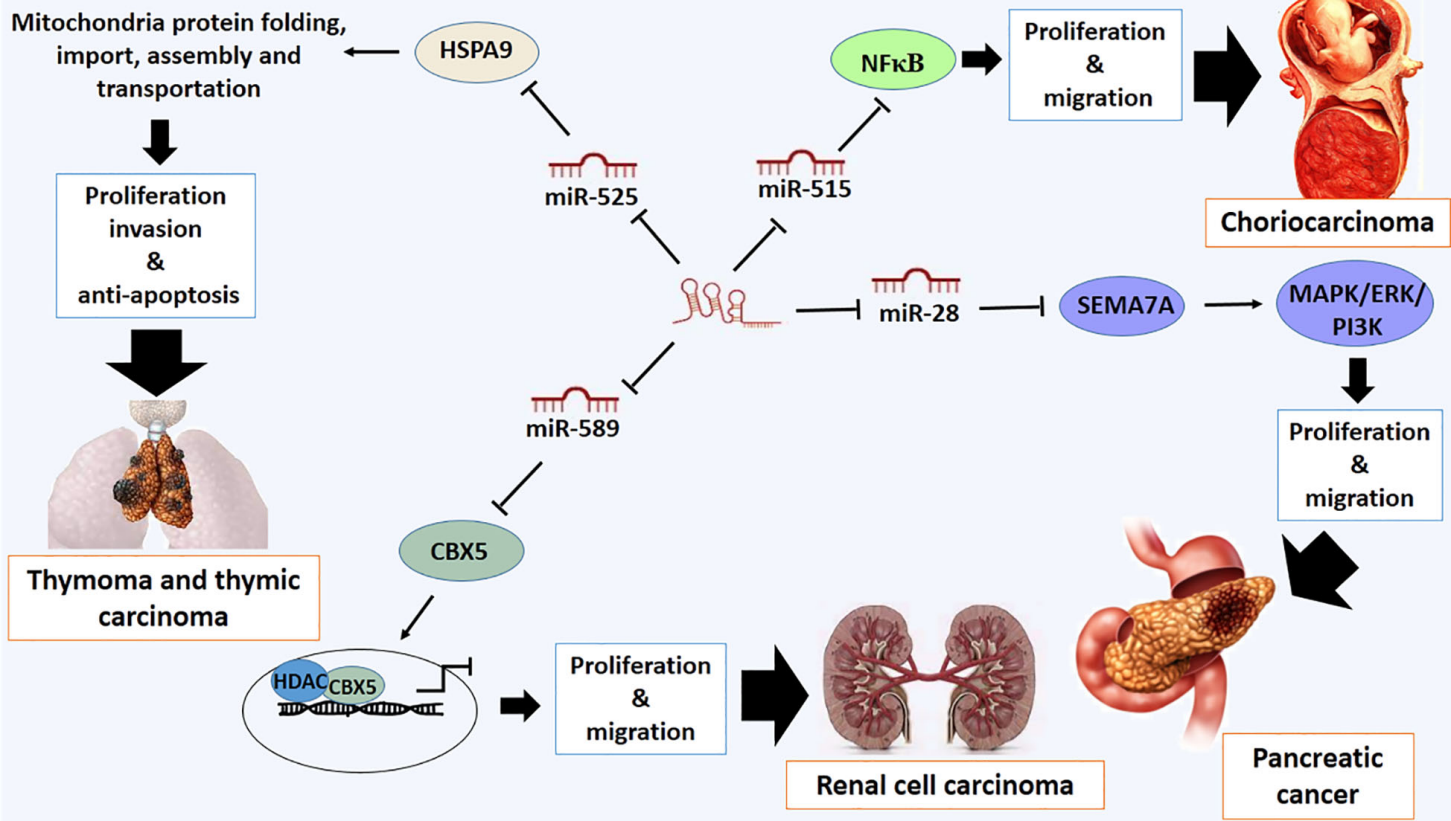
Molecular mechanistic activity of LncRNA LOXL1-AS1 in pancreatic cancer, choriocarcinoma, thymoma and thymic carcinoma and renal cell carcinoma.

Deregulation of LOXL1-AS1 has also been reported in some types of other cancer such as pancreatic cancer, osteosarcoma, medulloblastoma, choriocarcinoma, retinoblastoma, thymoma and thymic carcinoma and renal cell carcinoma (RCC) (23, 32, 98–102). It has been confirmed that LOXL1-AS1 exhibits a crucial role in pancreatic cancer development through miR-28–5p (98). One of the direct targets of miR-28–5p is Semaphorin 7A (SEMA7A), which promotes the proliferation and migration of cancer cells by regulating integrin-mediated signaling pathways and ERK activation (103, 104). Functionally, SEMA7A is a member of the semaphorin family of signaling proteins, which is anchored to cell membranes via glycosylphosphatidylinositol. The interaction of SEMA7A to β1-integrin triggers downstream signaling cascades, including MAPK/ERK and PI3K/AKT pathways (105). The overexpression of SEMA7A can neutralize the silenced expression of LOXL1-AS1 by increasing the proliferation rate of pancreatic cancer cells. Therefore, LOXL1-AS1 and miR-28–5p negatively regulate miR-28–5p and SEMA7A, respectively (98) (**Figure 6**).

LOXL1-AS1 is also expressed at a high level in osteosarcoma (99, 106). There is a positive association between the expression level of LOXL1-AS1 and tumor size, stage, and grade, distant metastasis, and survival time in osteosarcoma patients. It has been demonstrated that LOXL1-AS1 overexpression promotes cell proliferation, migration, and invasion by regulating phospho-PI3K and phospho-AKT (pAKT) expression, thereby activating the PI3K-AKT pathway in osteosarcoma (99).

Furthermore, LOXL1-AS1 is overexpressed in advanced stages of medulloblastoma, which is the most common type of brain tumor with a malignant phenotype in childhood (23). The knockdown of LOXL1-AS1 has been linked to cell cycle arrest at the G2/M phase, inducing apoptosis, inhibiting migration, and suppressing epithelial-mesenchymal transition (EMT) (23). Also, it has been associated with decreasing phosphorylated PI3K and AKT. Phosphorylation of PI3K and AKT activates and phosphorylates the downstream molecules in PI3K/AKT signaling pathways. Therefore, it is proposed that LOXL1-AS1 may be involved in the regulation of proliferation and apoptosis through initiation of the PI3K-AKT pathway in medulloblastoma (23). Also, it has been reported that applying LOXL1-AS1 siRNA-loaded exosomes can be considered as novel strategy for LOXL1-AS1 gene therapy in this type of cancer that leads to inhibit cancer progression and metastasis of medulloblastoma (107).

Additionally, the overexpression of LOXL1-AS1 stimulates the proliferation and migration of human choriocarcinoma cells through modulation of the nuclear factor kappa B (NFκB) signalling pathway (100). The role of LOXL1-AS1 on the NFκB signaling pathway may be mediated by targeting miR-515–5p. This miRNA, as a tumor-suppressive miRNA, regulates the NF-κB signaling pathway by decreasing the expression of phosphorylated p65 (p-p65) and phosphorylated IκBα (p-IκBα), which ultimately leads to the suppression of proliferation and migration of human choriocarcinoma cells. Therefore, it is recommended that the LOXL1-AS1/miR-515–5p/NF-κB signaling pathway is involved in the progression of human choriocarcinoma (100) (**Figure 6**).

High expression of LOXL1-AS1 has also been observed in retinoblastoma tumors (101). Retinoblastoma (RB) is an uncommon type of eye cancer that affects the retina. It primarily affects young children and can be hereditary or non-hereditary (108). LOXL1-AS1 may modulate the development of regulatory B (RB) cells, proliferation, migration, and metastasis by regulating the mitogen-activated protein kinase (MAPK) signalling pathway (101).

Thymomas and thymic carcinomas are both types of cancer that arise from the thymus gland (109). Thymoma is a relatively rare cancer that arises from the cells of the thymus. It typically grows slowly and is often found in adults, though it can also occur in children. Thymic carcinoma tends to grow and spread more quickly than thymoma. Most patients are typically diagnosed at a late stage of the disease because there are no specific symptoms in the early stages of these tumors (109). There is a positive correlation between the expression levels of LOXL1AS1 and HSPA9, and there is a negative association between miR-525–5p and HSPA9 in thymoma and thymic carcinoma. High expression levels of LOXL1-AS1 and *HSPA9* and downregulation of miR-525-5p have been observed in these types of tumors (102). It suggests that LOXL1-AS1 sponges miR-525–5p, and miR-525–5p targets the 3’ untranslated region (3’ UTR) of *HSPA9* mRNA directly. Therefore, LOXL1-AS1 promotes proliferation and invasion and suppresses apoptosis in both thymoma and thymic carcinoma (102). HSPA9, also known as mortalin or heat shock protein 70 (mtHsp70), is a chaperone protein located in the mitochondria, where it plays a crucial role in protein folding, importation, assembly, and transportation. The protein is involved in various cellular processes, including protection against stress and regulation of apoptosis (110). Mortalin binds to p53 and inhibits the translocation of p53 from the cytosol to the nucleus to act as a transcription factor and tumor suppressor protein (110). Therefore, the overexpression of HSPA9, resulting from the overexpression of LOXL1AS1, inhibits apoptosis and promotes proliferation and invasion in both thymoma and thymic carcinoma (**Figure 6**).

Overexpression of Lox1-AS1 has also been observed in RCC. Lox1-AS1 is implicated in cell proliferation and migration of these types of cancer cells, which occurs through miR-589–5p, whose expression levels are low in RCC (111). Tumor-suppressive activity of miR-589–5p is mediated by targeting Chromobox protein 5 (CBX5), which is recognized as an oncogene, accelerating proliferation and migration in RCC (111). CXB5 is a member of the chromatin assembly factor and chromatin remodeling complex that plays a role in epigenetic regulation and chromatin organization (112). CXB5 is involved in various procedures such as gene silencing, DNA repair and regulation of cell proliferation and metastasis of cancer cells (112). It has been shown that the oncogenic activity of CBXs may be mediated by recruiting histone deacetylases (HDAC). However, the role of CBX5 in the development and progression of RCC remains unknown (113). It has been shown that CBX5 rescue or miR-589–5p silencing can reverse the suppressive effects of silenced LOXL1-AS1 on the malignant phenotype of RCC (111) (**Figure 6**).

**Table 1** presents direct targets and molecular mechanistic activity of LOXL1-AS1 in different cancer cells.

**Table 1:** Direct targets and molecular mechanistic activity of LOXL1-AS1 in different cancer cells.

Type of cancer	Experimental samples/models	Direct target	Tumorigenicity activity	References
Ovarian cancer	Cell lines, Xenograft mouse Cell lines, tissues	miR-761 miR-18b-5p/*VMA21* axis	Proliferation and anti-apoptosis Proliferation and metastasis	(30) (43)
Cervical cancer	Cell lines Cell lines, tissues Cell lines, tissues, Xenograft mouse	miR-21/RHOB axis miR-526b-5p/LYPLA1 axis miR-423–5p/ENC1 axis	Proliferation and migration Proliferation, migration, invasion, and angiogenesis Proliferation and metastasis	(47) (49) (52)
Endometrial cancer	Cell lines, tissues, Xenograft mouse	miR-28–5p/RAP1B axis	Proliferation, migration, invasion and anti-apoptosis	(54)
Gastric cancer	Cell lines, tissues, Xenograft mouse Cell lines, tissues, Xenograft mouse	miR-708–5p/USF1 axis miR-142–5p/PIK3CA axis	Proliferation, migration, EMT and stemness phenotypes Proliferation, migration and metastasis	(31) (58)
Colorectal cancer	Cell lines, tissues Cell lines, tissues, Xenograft mouse Cell lines, tissues, Xenograft mouse	miR-708–5p/CD44/EGFR axis miR-1224–5p/HK2 axis miR-761/HK2 axis	Proliferation, migration and invasion Proliferation, invasion, migration, glycolysis and anti-apoptosis Proliferation, invasion, migration, glycolysis and anti-apoptosis	(32) (60) (60)
Esophageal squamous cell carcinoma	Cell lines, tissues	DESC1	Proliferation, migration, invasion and anti-apoptosis	(62)
Lung cancer	Cell lines, tissues Cell lines, tissues Cell lines, tissues	miR-324–3p miR-423–5p/MYBL2 axis miR-3128/RHOXF2 axis	Proliferation and invasion Proliferation and anti-apoptosis Metastasis	(33) (38) (65)
Laryngeal carcinoma	Cell lines, Xenograft mouse	miR-589–5p/TRAF6 axis	Proliferation, migration, and EMT	(73)
Hepatocellular carcinoma	Cell lines Cell lines Cell lines, tissues	miR-1224–5p/ITPRIPL2 axis miR-3614–5p/YY1 axis miR-377–3p/NFIB axis	Proliferation, migration and invasion Proliferation, migration, invasion and anti-apoptosis Proliferation, migration and EMT	(34) (80) (82)
Cholangiocarcinoma	Cell lines, tissues	miR-324–3p/ABCA1 axis	Proliferation, migration, invasion and anti-apoptosis	(37)
Breast cancer	Cell lines, tissues Cell lines, tissues Cell lines, tissues	miR-143–3p miR-708–5p EZH2	Proliferation, migration, invasion and anti-apoptosis Invasion and metastasis Invasion and metastasis	(35) (88) (88)
Prostate cancer	Cell lines Doxorubicin-resistant cell line, Xenograft mouse	miR-541–3p/CCND1 axis miR-let-7a-5p/EGFR	Proliferation Proliferation, migration and anti-apoptosis	(36) (91)
Glioblastoma	Cell lines Cell lines, tissues, Xenograft mouse	RelB miR-374b-5p/MMP14 axis	Mesenchymal phenotype Proliferation, migration, invasion and vasculogenic mimicry	(94) (97)
Pancreatic cancer	Cell lines	miR-28–5p/SEMA7A axis	Proliferation and migration	(98)
Choriocarcinoma	Cell lines	miR-515–5p	Proliferation and migration	(100)
Thymoma and thymic carcinoma	Cell lines, tissues	miR-525-5p/HSPA9 axis	Proliferation and invasion and anti-apoptosis	(102)
Renal cell carcinoma	Cell lines, tissues	miR-589–5p/CBX5 axis	Proliferation and migration	(111)

**Figure 7:**
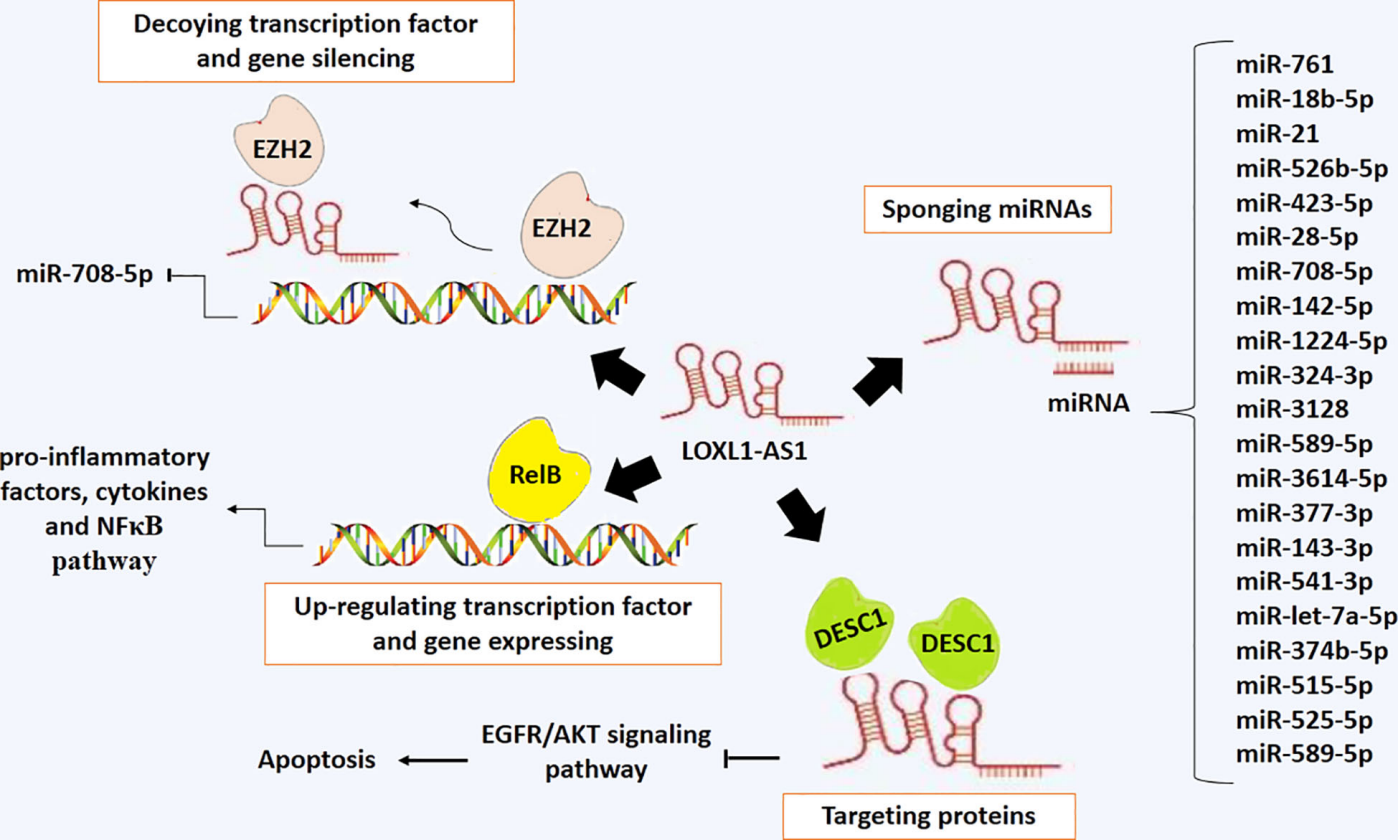
The regulatory mechanisms of LOXL1-AS1, which include miRNA sponging, protein targeting, gene silencing, and gene expression.

**Figure 7** presents the regulatory mechanisms of LOXL1-AS1, which include miRNA sponging, protein targeting, gene silencing, and gene expression.

## Comparing LOXL1-AS1 with other oncogenic lncRNAs

LncRNAs are categorized into two groups: oncogenic and tumor-suppressive lncRNAs. LOXL1-AS1 has been studied mainly as an oncogenic lncRNA, implicated in tumor growth and development through sponging tumor suppressor miRNAs and, or interacting with proteins and transcription factors such as DESC1 and EZH2. Many long non-coding RNAs (lncRNAs) having clinical relevance for therapy and diagnosis have been introduced as oncogenic lncRNAs, including MALAT1, HOTAIR, PCA3, CCAT1, CCAT2, H19, HOTTIP, and UCA1 (114). Similar to these oncogenic lncRNAs, LOXL1-AS1 induces malignant and metastatic properties, and it has been introduced as a potential therapeutic and diagnostic option. Like LOXL1-AS1, other lncRNAs, such as HOTAIR, PCA3, HOTTIP, and UCA1, have mainly exhibited oncogenic activities (115–118), while the others including MALAT1, CCAT1, CCAT2 and H19 have evidence exhibiting both oncogenic and tumor-suppressive activities (119–121). So far, the tumor-suppressive role of LOXL1-AS1 has not been confirmed in cancer cell lines or cancer tissues, whereas all studies conducted to date have indicated its oncogenic role; thus, LOXL1-AS1 may prove valuable as a diagnostic biomarker or therapeutic target.

## Technologies for identifying lncRNAs activity in various disease

LncRNAs exhibit tissue-specificity and generally possess low expression levels, necessitating the selection of suitable experimental approaches for their detection and analysis. To study lncRNA activity in different diseases, various technologies have been developed to characterize their functions and mechanisms of action (122–126).

One of the key technologies used to study long non-coding RNA (lncRNA) activity is RNA sequencing, which enables researchers to identify and quantify lncRNAs in various tissues and cell types. By comparing the expression levels of lncRNAs in healthy and diseased samples, researchers can gain insights into the role of lncRNAs in disease development and progression. In addition, RNA sequencing can also identify lncRNAs and their interactions with other biomolecules (122).

Another important technology used to study lncRNA activity is chromatin immunoprecipitation sequencing (ChIP-seq), which allows researchers to identify the binding sites of lncRNAs on chromatin. By mapping the genomic locations of lncRNAs, researchers can determine their regulatory targets, providing insights into the mechanisms by which lncRNAs regulate gene expression. ChIP-seq data can also identify protein partners of lncRNAs, further elucidating their functional roles (122). Also, chromatin isolation by RNA purification followed by high-throughput sequencing (ChiRP-seq) is a technique used to study the interactions between lncRNAs and genomic DNA, or other DNA-binding proteins. The lncRNA of interest is selectively purified along with its associated DNA and proteins using complementary antisense DNA oligonucleotides to pull down the RNA complexes. The isolated RNA, DNA, or protein is then sequenced to identify the specific genome loci and associated proteins that interact with the lncRNA. This technique has been instrumental in uncovering the functional roles of lncRNAs in gene regulation and chromatin organization (123).

In addition to RNA sequencing and ChIP-seq, researchers use CRISPR/Cas9-based genome editing to study lncRNA activity. By targeting specific lncRNAs with CRISPR/Cas9, researchers can investigate the effects of lncRNA knockdown or overexpression on gene expression and cellular functions. This technology allows for the precisely manipulating lncRNA activity, providing valuable insights into their regulatory roles in disease (127).

Moreover, CRISPR interference (CRISPRi) and CRISPR activation (CRISPRa) technologies have been developed to study the functional roles of lncRNAs in gene regulation. CRISPRi allows researchers to selectively silence lncRNA expression, while CRISPRa enables the activation of specific lncRNAs in a targeted manner (123, 127).

Additionally, ribonucleoprotein immunoprecipitation sequencing (RIP-seq) has been widely used to study the interactions between lncRNAs and RNA-binding proteins. By immunoprecipitating lncRNA-protein complexes and sequencing the associated RNA molecules, researchers can identify the protein partners of lncRNAs and their functional interactions. RIP-seq data can provide insights into the molecular mechanisms by which lncRNAs regulate gene expression and cellular processes in diseases (126).

Apart from these technologies, high-throughput screening methods, such as CRISPR knockout screens and RNA interference (RNAi) screens, have been used to identify lncRNAs involved in specific disease pathways. By systematically knocking down or silencing lncRNAs in cell populations, researchers can identify those that play crucial roles in disease pathogenesis. These screens can also elucidate the functional relationships between lncRNAs and protein-coding genes, providing insights into disease mechanisms (124).

In addition, the RNA fluorescence *in situ* hybridization (FISH) technique has been employed for several years to locate specific RNA molecules within cells. This method relies on the hybridizing specifically designed fluorescently labeled probes to their target sequences. However, traditional RNA Fish lacks sufficient sensitivity to detect low-abundance long non-coding RNA (lncRNA) molecules. To address this limitation, a short molecular beacon-based RNA fluorescence *in situ* hybridization (smRNA-FISH) approach has been developed. smRNA-Fish utilizes a pool of short probes that cover the entirety of lncRNAs, enabling highly sensitive detection of these low-abundance transcripts while also providing quantifiable measurements (124).

Furthermore, bioinformatics tools (LncFinder, lncRNA-LSTM, LncPred-IEL, PredLnc-GFStack, RNAplonc, NCResNet, …) and databases (CSG, GermlncRNA, LNCat, LncSNP, Lnc2Cancer, lnCeDB, LNCMap, Lnc2Meth, lncATLAS, lncPedia, lncRNAdisease, lncRNome, …) have been developed to analyze and interpret lncRNA data generated from various technologies (128, 129). These tools allow researchers to integrate multi-omics data, predict lncRNA functions, and identify potential therapeutic targets (129). By combining experimental approaches with computational analyses, researchers can gain a comprehensive understanding of long non-coding RNA (lncRNA) activity in various diseases and develop novel strategies for diagnosis and treatment.

## Conclusion

It has been proven that lncRNAs have both oncogenic and tumor-suppressive roles in regulating molecular mechanisms involved in cancer progression, including proliferation, migration, metastasis, and EMT. LOXL1-AS1 is a novel recognized lncRNA, which is well known as an oncogenic lncRNA. LOXL1-AS1 is overexpressed in a variety of cancer cells, including ovarian cancer, gastrointestinal cancers, lung cancer, hepatocellular carcinoma, breast cancer, and prostate cancer, and others. It can also regulate malignant phenotypes of these types of cancer cells by targeting specific miRNAs such as miR-761, miR-423–5p, miR-21, miR-28–5p, miR-708–5p, miR-423–5p, etc. and prevent miRNAs to binding to their target mRNAs and thereby regulate gene expression, indirectly. In addition, some common miRNAs (miR-761, miR-423–5p, miR-28–5p, miR-708–5p, miR-1224–5p, miR-324–3p) are sponged by LOXL1-AS1 in various types of cancer cells (**Table 2**). LOXL1-AS1 targets miR-761, miR-708–5p and miR-1224–5p in colorectal cancer cells. miR-761 and miR-1224–5p, which are both sponge targets of LOXL1-AS1, share a common target gene, HK2, in colorectal cancer. miR-761, miR-708–5p, and miR-1224–5p are also downregulated in ovarian cancer, gastric cancer, and hepatocellular carcinoma, respectively. Both miR-423–5p and miR-324–3p are sponged by LOXL1-AS1 in lung cancer. miR-423–5p and miR-324–3p are also suppressed in cervical cancer and cholangiocarcinoma, respectively. miR-28 is suppressed by LOXL1-AS1 in both pancreatic and endometrial cancers.

**Table 2:** Common miRNAs sponged by LOXL1-AS1 in several types of cancer.

miRNA	Target gene	Type of cancer	Reference
miR-761	HK2	Colorectal cancer Ovarian cancer	(60) (30)
miR-423–5p	MYBL2 ENC1	Lung cancer Cervical cancer	(38) (52)
miR-28–5p	SEMA7A RAP1B	Pancreatic cancer Endometrial cancer	(98) (54)
miR-708–5p	USF1 CD44/EGFR	Gastric cancer Colorectal cancer Breast cancer	(31) (32) (88)
miR-1224–5p	ITPRIPL2 HK2	Hepatocellular carcinoma Colorectal cancer	(34) (60)
miR-324–3p	ABCA1	Cholangiocarcinoma Lung cancer	(37) (33)

On the other hand, LOXL1-AS1 can interact with several proteins, including DESC1, EZH2, and EGFR, and modulate their activities. Therefore, there is a diversity and different molecular mechanisms in the oncogenic activity of LOXL1-AS1 based on the cancer cell type.

Knowledge about the molecular mechanisms regulated by LOXL1-AS1 in cancer cells can open up ways to identify specific prognostic biomarkers and discover novel therapeutic approaches for various types of cancer. Upregulation of LOXL1-AS1 has been confirmed in many types of cancerous tissues, which can exhibit a clinical value of LOXL1-AS1 and introduce it as a diagnostic biomarker. However, comprehensive clinical studies have not yet been conducted. There is still limited clinical evidence to investigate the association between the expression of LOXL1-AS1 and clinical features such as tumor size, stage and grade, distant metastasis, and survival time of patients. Further studies are needed to fully evaluate the clinical significance of LOXL1-AS1 and confirm its potential for use as a diagnostic tool, as well as exploring LOXL1-AS1 as a novel therapeutic approach for treating various types of cancer.

## Author contributions

SY: Conceptualization, Data curation, Investigation, Methodology, Project administration, Resources, Software, Supervision, Validation, Visualization, Writing – original draft, Writing – review & editing.

## Glossary

ABCA1: ATP-binding cassette transporter A1
CSCC: Cervical squamous cell carcinoma
CCA: Cholangiocarcinoma
ChIP-seq: Chromatin immunoprecipitation sequencing
ChiRP-seq: Chromatin isolation by RNA purification followed by high-throughput sequencing
CBX5: Chromobox
CeRNAs: Competing endogenous RNAs
CRISPRa: CRISPR activation
CRISPRi: CRISPR interference
CCND1: Cyclin D1
DESC1: Differentially expressed in squamous cell carcinoma 1
ENC1: Ectodermal-neural cortex 1
EC: Endometrial cancer
EZH2: Enhancer of zeste homolog 2
EGFR: Epithelial growth factor receptor
EMT: Epithelial-mesenchymal transition
ESCC: Esophageal squamous cell carcinoma
FISH: Fluorescence *in situ* hybridization
HSP70: Heat shock protein 70
HCC: Hepatocellular carcinoma
HK2: Hexokinase 2
HDAC: Histon deacetylases
IP3R: Inositol 1,4,5-trisphosphate receptor
ITPRIPL2: Inositol 1, 4, 5-trisphosphate receptor-interacting protein-like 2
LOXL1-AS1: LncRNA LOXL1-antisense RNA 1
LncRNAs: Long non coding RNAs
LYPLA1: Lysophospholipase 1
MMPs: Matrix metalloproteases
MEK/ERK: Mitogen-activated protein kinase/extracellular signal-regulated kinase
MREs: miRNA response elements
MSI1: Musashi1
MYBL2: Myb-related protein B
NRDR: Non-coding RNA Database Resource
NCAPH: Non-SMC Condensin I Complex Subunit H
NSCL: Non-small-cell lung cancer
NFIB: Nuclear factor I B
RAP1B: Ras-related protein1B
RSR: Relative survival rate
RCC: Renal cell carcinoma
RB: Retinoblastoma
RHOXF2: Rhox homeobox family member 2
RIP-seq: Ribonucleoprotein immunoprecipitation sequencing
RNAi: RNA interference
SEMA7A: Semaphorin 7A
smRNA-Fish: Short molecular beacons-based RNA fluorescence *in situ* hybridization
TIAR: TIA-1 related protein
TRAF6: Tumor necrosis factor receptor-associated factor 6
USF1: Upstream transcription factor 1
VMA21: Vacuolar ATPase Assembly Factor
VM: Vasculogenic mimicry
YY1: Yin Yang 1
